# Fibroblast growth factor 23, mineral metabolism and mortality among elderly men (Swedish MrOs)

**DOI:** 10.1186/1471-2369-14-85

**Published:** 2013-04-15

**Authors:** Per-Anton Westerberg, Åsa Tivesten, Magnus K Karlsson, Dan Mellström, Eric Orwoll, Claes Ohlsson, Tobias E Larsson, Torbjörn Linde, Östen Ljunggren

**Affiliations:** 1 Department of Medical Sciences, Uppsala University, Uppsala, Sweden; 2 Wallenberg Laboratory for Cardiovascular Research, University of Göteborg, Göteborg, Sweden; 3 Clinical and Molecular Osteoporosis Research Unit, Department of Clinical Sciences and Orthopedic Surgery, Lund University, Skåne University Hospital, Lund, Sweden; 4 Center for Bone and Arthritis Research at the Sahlgrenska Academy, Institute of Medicine, The Sahlgrenska Academy at Göteborg University, Göteborg, Sweden; 5 Oregon Health and Science University, Portland, OR, USA; 6 Department of Clinical Science, Intervention and Technology, Karolinska Institute, Stockholm, Sweden; 7 Department of Nephrology, Uppsala University, University hospital, Ing 30, 5 tr, Uppsala, SE-751 85, Sweden

**Keywords:** FGF-23, FGF23, Mineral metabolism, Phosphatonin, Mortality, Elderly

## Abstract

**Background:**

Fibroblast growth factor 23 (FGF23) is the earliest marker of disturbed mineral metabolism as renal function decreases. Its serum levels are associated with mortality in dialysis patients, persons with chronic kidney disease (CKD) and prevalent cardiovascular disease (CVD), and it is associated with atherosclerosis, endothelial dysfunction and left ventricular hypertrophy in the general population. The primary aim of this study is to examine the association between FGF23 and mortality, in relation to renal function in the community. A secondary aim is to examine the association between FGF23 and CVD related death.

**Methods:**

The population-based cohort of MrOS Sweden included 3014 men (age 69–81 years). At inclusion intact FGF23, intact parathyroid hormone (PTH), 25 hydroxyl vitamin D (25D), calcium and phosphate were measured. Mortality data were collected after an average of 4.5 years follow-up. 352 deaths occurred, 132 of CVD. Association between FGF23 and mortality was analyzed in quartiles of FGF23. Kaplan-Meier curves and Log-rank test were used to examine time to events. Cox proportional hazards regression was used to examine the association between FGF23, in quartiles and as a continuous variable, with mortality. The associations were also analyzed in the sub-cohort with estimated glomerular filtration rate (eGFR) above 60 ml/min/1.73 m^2^.

**Results:**

There was no association between FGF23 and all-cause mortality, Hazard ratio (HR) 95% confidence interval (CI): 1.02 (0.89-1.17). For CVD death the HR (95% CI) was 1.26 (0.99 - 1.59)/(1-SD) increase in log(10)FGF23 after adjustment for eGFR, and other confounders. In the sub-cohort with eGFR > 60 ml/min/1.73 m^2^ the HR (95% CI) for CVD death was 55% (13–111)/(1-SD) increase in log(10)FGF23.

**Conclusions:**

FGF23 is not associated with mortality of all-cause in elderly community living men, but there is a weak association with CVD death, even after adjustment for eGFR and the other confounders. The association with CVD death is noticeable only in the sub-cohort with preserved renal function.

## Background

Decreased renal function, defined as estimated glomerular filtration rate (eGFR) below 60 ml/min/1.73 m^2^, is an independent risk factor for mortality of cardiovascular disease (CVD), not fully explained by traditional risk factors. Serum concentration of fibroblast growth factor 23 (FGF23) increases in parallel with parathyroid hormone (PTH) as renal function declines in chronic kidney disease (CKD) to maintain phosphate and calcium homeostasis [1,2]. In an elderly population mild to moderate renal dysfunction is common and implies an increased risk for CVD related mortality [3]. Hyperphosphatemia, increased PTH and FGF23 have been associated with CVD risk in observational studies [4-6]. Also higher phosphate levels in the normal range is associated with increased risk for CVD death in individuals with prevalent coronary disease [4].

FGF23 may be a better indicator of phosphate exposure, and CVD risk, in subjects with mild to moderate renal dysfunction due to that hyperphosphatemia does not develop until advanced renal dysfunction, serum phosphate is subject to circadian variation and may vary with intake, glucose metabolism and anabolic status. Actually, FGF23 is associated with left ventricular hypertrophy [7,8], extent of atherosclerosis [9], endothelial dysfunction [10], and increased risk for mortality in CKD [6] and in those with prevalent coronary disease [11].

Serum concentration of 25 hydroxyl vitamin D (25D), which is the precursor of active vitamin D, 1,25 dihydroxy vitamin D (1,25D), reflects the vitamin D status of the individual. The 1,25D level is precisely regulated by PTH and FGF23, which have opposite effects on its activation in the kidney. 25D deficiency induces an increase in PTH. Lower levels 25D are associated with mortality in observational studies [12-14].

We hypothesize that disturbed mineral metabolism is one explanation for the increased mortality risk associated with decreased eGFR, and that FGF23, as a potentially modifiable marker of phosphate load, is independently associated with mortality risk in an elderly community derived population.

The primary aim of the present investigation is to study the relationship between FGF23 and mortality, in relation to the other parameters of mineral metabolism. The secondary aim is to study the relationship between FGF23 and CVD related mortality. We will also report the associations between PTH and 25D with mortality.

## Methods

### Study population

The multicenter prospective Osteoporotic Fractures in Men (MrOS) study which included older men in Sweden, Hong Kong, and the United States aimed at examine aspects of male osteoporosis and healthy ageing. We used the participants in MrOS Sweden for this study. Participants (men aged 69–81 years) were randomly selected from national population register and invited by letter. 45% of those contacted participated (n = 3014), constituting three sub cohorts in three cities: Malmoe (n = 1005), Gothenburg (n = 1010) and Uppsala (n = 999). Eligibility for study participation required the ability to walk unassisted, to provide self-reported data, and to understand and sign an informed consent. The ethics committees at the Universities of Gothenburg (approval number Gbg M 014–01), Malmoe (LU-693-00), and Uppsala (Ups 01–057) approved of the study, which were done in accordance with the declaration of Helsinki.

### Assessment of parameters of mineral metabolism

Serum samples were collected after at least 10 hours of fasting and stored at −80°C. Serum intact FGF23 was analyzed with a two-site monoclonal antibody based ELISA (Kainos Laboratories International; Tokyo, Japan). The assay has a lower limit of detection of 3 ng/L. Intra-assay coefficient of variation was less than 5% and inter-assay coefficient of variation was 6%. Levels of FGF23 were available for 97% of the participants in the Gothenburg cohort, 94% in the Malmoe cohort, and 91% in the Uppsala cohort. Levels between 0 and 3 (N = 18) was given the value 1.5 ng/L, and levels above 800 (N = 2) were given the value 800 ng/L.

Intact PTH was measured by a second generation immunometric assay, Immulite 2000, (Los Angeles, USA).

Serum PTH and FGF23 had right skewed distributions and were log(10)-transformed to approximate normality. When analyzed as a continuous variable they were further z-transformed to allow reporting the results/(1-standard deviation (SD)) change. 25D levels were measured by Nichols Advantage automated assay system (San Juan Capistrano, CA, USA).

### Assessment of mortality

Mortality data were collected from the population statistics at Statistics Sweden. Follow-up time was recorded as the period between baseline visit (in 2001–2004) and date of death or mortality data collection (March 1, 2008). Cause of death data were collected from the Swedish Cause of Death Register, held by the National Board of Health and Welfare in Sweden, in which all deaths in Sweden are registered with International Classification of Diseases (ICD) codes based on the information from death certificates. The data were collected from this register from the study start until the last update of the register December 31, 2005 and from evaluation of copies of death certificates for deaths occurring after this date up to 2008. Based on the information from the register or death certificates, the underlying death cause was determined for each participant and CVD death was defined by ICD-10 codes I00 to I99.

### Assessment of covariates

A standardised questionnaire was used to gather information about smoking and self-reported medical diagnoses (hypertension, diabetes, cancer, myocardial infarction, angina pectoris, and stroke). Prevalent CVD was defined as a history of myocardial infarction, angina pectoris or stroke. Hypertension was defined as self-reported anti-hypertensive treatment for diagnosed hypertension or a systolic blood pressure above 140 mmHg measured once on inclusion (supine blood pressure, measured after 10 minutes rest). Body mass index (BMI) was calculated as weight/height^2^ (kg/m^2^).

Phosphate, calcium and albumin were analyzed at respective hospitals department for clinical chemistry using standard methods. We report calcium and albumin as separate variables. Estimated glomerular filtration rate (eGFR) in ml/min/1.73 m^2^ was calculated from serum cystatin C (Cystatin C Immunoparticles, Dako A/S, Glostrup, Denmark) according to the formula 79.901*(Cyst C [mg/L])^-1.4389^[15].

### Statistical analysis

The baseline variables are presented as mean ± SD for normally distributed variables and as median (interquartile range (IQR) 25th, 75th percentiles) for non-normally distributed variables for the whole cohort and in quartiles of FGF23. Mortality rates are reported as deaths per 1000-person-years.

Spearman correlation coefficients were calculated to describe univariate correlations between FGF23, BMI, eGFR, calcium, phosphate, PTH, and 25D.

Kaplan Meier curves were created for quartiles of FGF23 showing proportion surviving all-cause death and CVD related death. Differences between quartiles were examined with Log rank test.

Cox proportional hazards regression was used to study the association between mortality (all-cause and CVD-cause) and FGF23, in quartiles and as a continuous variable after adjustment for confounders. Associations between mortality and log (10)PTH and 25D were also analyzed. Proportional hazard assumptions were confirmed by inspecting Schoenfeld residuals and linearity assumptions by inspecting Martingales residuals.

The models were adjusted for potential confounders, phosphate, calcium, albumin, PTH, 25D, eGFR and BMI, as well as smoking, prevalent diabetes mellitus, hypertension, cancer and prevalent CVD (coronary artery disease or stroke).

Age was included as a covariate in the first model. eGFR and phosphate were included in the second model, as they were judged the most relevant confounders based on biological plausibility as both eGFR and phosphate potentially affects FGF23 and mortality risk but are not in the causal pathway. In the third model all available covariates, including 25D, log(10)PTH and log(10)FGF23, where appropriate, and prevalent disorders, as well as BMI, were included to test if the association in model 2 changed substantially. All analyses were stratified for site. BMI and albumin were included as confounders since both are strong predictors for mortality and associated with parameters of mineral metabolism. As the GFR is an essential factor for all parameters of mineral metabolism we analyzed the sub-cohorts with eGFR above or below 60 ml/min/1.73 m^2^. We also analyzed those with, and without, prevalent CVD separately.

P < 0.05 was considered statistically significant. Statistical analyses were performed using Statistica Windows (version 10.0 StatSoft, Chicago, IL).

## Results

The characteristics of the whole cohort and quartiles based on serum FGF23 level are presented in Table 1. The mean (± SD) age was 75.5 ± 3.2 years and the eGFR was 72.0 ± 20 μmol/L, the median (IQR) for FGF23 was 43.5 (32.4 - 57.5) and the mortality rate (95% CI) was 27.7 (24.9 - 30.7)/1000 py and the CVD related mortality rate was 10.3 (8.5 - 12.0)/1000 py. With increasing quartile of FGF23 eGFR decreased, PTH increased and prevalence's of CVD and hypertension increased.

**Table 1:** Characteristics and biochemical parameters of the whole cohort and Quartiles: Q1 to Q4 of FGF23

**N = 2838**	**All**	**Q1**	**Q2**	**Q3**	**Q4**
	**N = 2838**	**N = 709**	**N = 710**	**N = 709**	**N = 710**
**FGF23 Range (pg/ml)**	1.5-800	1.5-32.2	32.4-43.4	43.5-57.4	57.5-800
**FGF23 median (IQR)(pg/ml)**	43.5 (32.4-57.5)	25.3 (19.2-28.8)	37.9 (35.3-40.4)	49.6 (46.5-53.0)	69.9 (62.7-85.5)
**Age (years)**	75.5 ± 3.2	75.5 ± 3.1	75.3 ± 3.2	75.5 ± 3.2	75.6 ± 3.2
**Weight (kg)**	80.6 ± 12.1	77.8 ± 11.3	80.7 ± 12.3*	81.1 ± 11.5*	82.9 ± 12.7*
**BMI (kg/m**^**2**^**)**	26.4 ± 3.6	25.6 ± 3.4	26.4 ± 3.6*	26.5 ± 3.3*	27.0 ± 3.8*
**Smoking (%)**	8	9	8	8	8
**Diabetes (%)**	9	7	10*	10*	10*
**Hypertension (%)**	36	30	33	36*	45*
**Prevalent CVD**	19	14	19*	19*	24*
**eGFR (ml/min/1.73 m**^**2**^**)**	72.0 ± 20	80.2 ± 18	75.2 ± 18*	71.1 ± 17*	61.5 ± 21*
**PTH median IQR (pmol/L)**	4.3 (3.0 - 5.8)	3.8 (2.7 - 5.1)	4.2* (3.0 - 5.8)	4.3* (3.0 - 5.8)	4.7* (3.2 - 6.4)
**Pi (mmol/L)**	1.07 ± 0.16	1.08 ± 0.16	1.06 ± 0.17*	1.07 ± 0.16	1.08 ± 0.16
**Ca (mmol/L)**	2.36 ± 0.16	2.39 ± 0.17	2.33 ± 0.16*	2.34 ± 0.14*	2.36 ± 0.15
**Albumin (g/L)**	43.1 ± 3.6	43.4 ± 3.7	43.5 ± 3.6	42.9 ± 3.6*	42.8 ± 3.5*
**25OHvitD (nmol/L)**	69.8 ± 23.6	70.6 ± 23.1	67.9 ± 23.3*	68.5 ± 22.4	72.2 ± 25.3
**Mortality rate/1000 py (95%CI)**	27.7 (24.9 - 30.7)	26.3 (20.8 - 31.8)	26.1 (20.6 - 31.7)	24.7 (19.2 - 30.1)	34.0 (27.9 - 41.0)
**Cardiovascular deaths/1000 py (95%CI)**	10.3 (8.5 - 12.0)	7.1 (4.3- 9.9)	12.2 (8.4- 15.9)	8.4 (5.2 - 11.6)	13.6 (9.5 - 17.8)

Descriptive statistics of the whole cohort and quartiles. IQR means interquartile range and is expressed as border for lowest quartile to highest quartile. BMI means body mass index and is calculated as weight in kg/(length in m) ^2^. CVD is cardiovascular disease, including coronary artery disease and stroke. * means differ from Q1, analysed by ANOVA for continuous normally distributed variables and by Kruskall-Wallis test for the others. P < 0.05 was considered significant.

Univariate correlations between parameters are presented in Table 2. FGF23 correlated with decreasing eGFR and, to some extent, calcium and PTH, but not with phosphate.

**Table 2:** Spearman correlation coefficients between parameters of mineral metabolism and BMI

**(N = 2838)**	**BMI**	**eGFR**	**Albumin**	**Calcium**	**Phosphate**	**25D**	**Log PTH**
**LogFGF23**	0.12 *	−0.35 *	−0.06 *	−0.06 *	0.001 ^N.S.^	0.03 ^N.S.^	0.19 *
**LogPTH**	0.03 ^N.S.^	−0.22 *	0.09 *	−0.36 *	−0.13 *	−0.19 *	
**25D**	−0.12 *	−0.006 ^N.S.^	0.02 ^N.S.^	0.13 *	0.04 *		
**Phosphate**	−0.05 *	−0.05 *	0.11 *	0.15 *			
**Calcium**	0.02 ^N.S.^	0.05 *	0.17 *				
**Albumin**	−0.01 ^N.S.^	0.07 *					
**eGFR**	−0.09 *						

BMI is body mass index, eGFR is estimated glomerular filtration rate, 25D is 25 hydroxy vitamin D, * means significant correlation between parameters (P < 0.05). N.S means non-significant.

**Figure 1:**
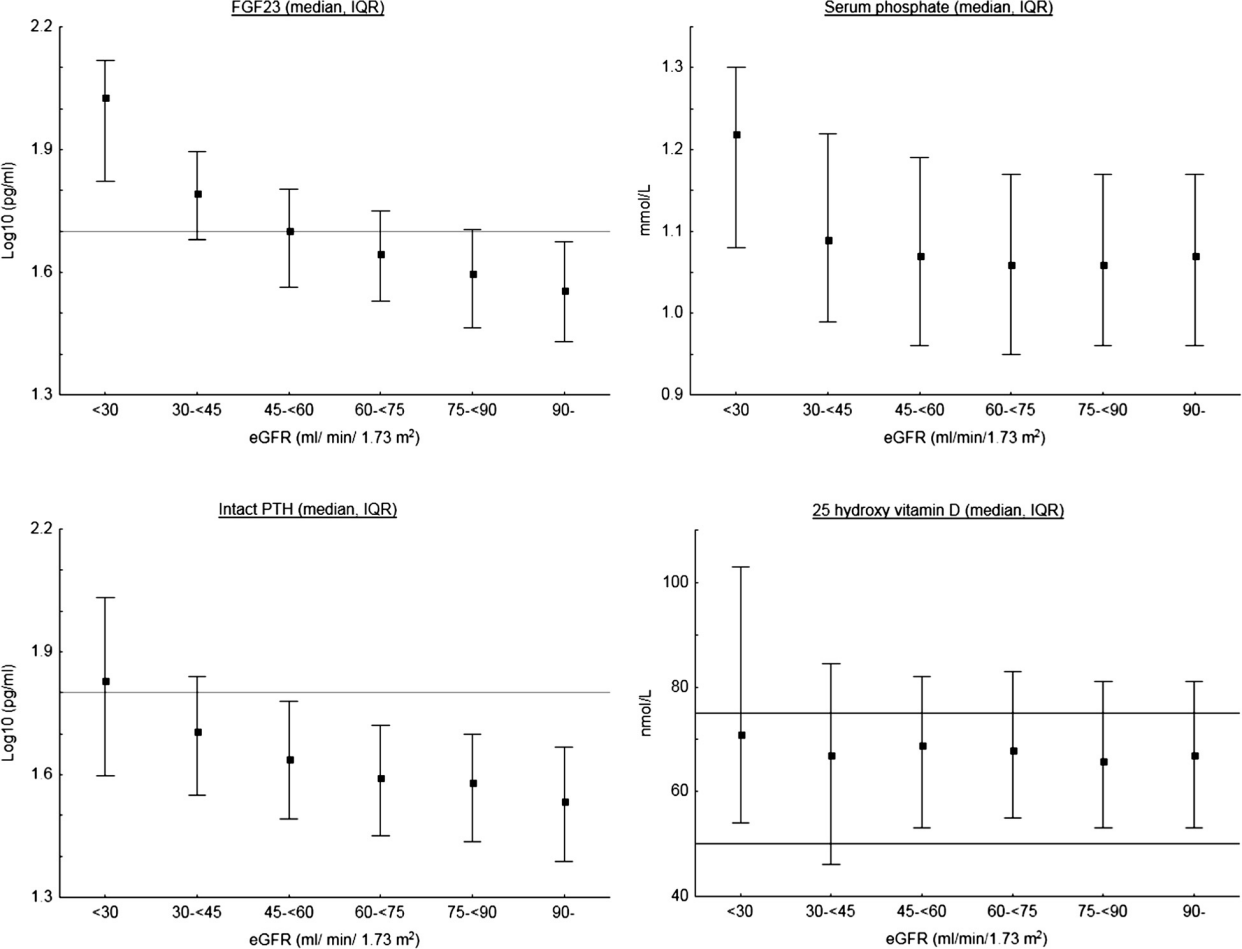
**LogFGF23, LogPTH, phosphate and 25 D in relation to eGFR <30 (N = 43), 30- < 45 (N = 181), 45- < 60 (N = 539), 60- < 75 (N = 848), 75- < 90 (N = 740) and 90- (N = 487).** 46 subjects excluded due to missing values. Reference lines indicate 50 pg/ml for FGF23, 65 pg/ml for PTH and 75 and 50 nmol/L for 25 hydroxyl vitamin D (25D). To convert PTH to pmol/L multiply by 0.11, phosphate to mg/dL multiply by 3.125 and 25D to ng/ml multiply by 0.4.

The parameters of mineral metabolism are depicted in relation to eGFR in Figure 1. As eGFR decrease, FGF23 and PTH increase in parallel. There is a detectable increase in FGF23 before PTH, as more than 25% of participants with eGFR between 75 and 90 ml/min/1.73 m^2^ had FGF 23 levels above 50 ng/L, while over 75% had PTH levels below 65 ng/L until eGFR was below 30 ml/min/1.73 m^2^. Phosphate did not increase until eGFR was below 30 ml/min/1.73 m^2^. There was no correlation between 25D levels and eGFR and the majority of participants had levels between 50 and 75 nmol/L.

**Figure 2:**
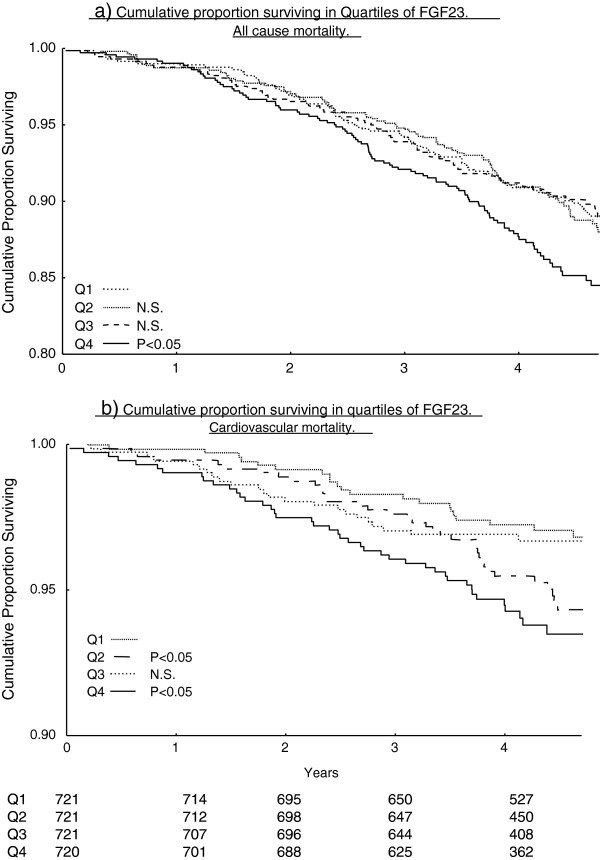
**Kaplan-Meier curves of proportional survival due to all-cause (2a) and cardiovascular (2b) death for quartiles of FGF23.** P means probability for non difference to Q1 examined by Log-rank test.

Kaplan-Meier curves for quartiles of FGF23 and all-cause mortality (Figure 2a) and CVD related mortality are shown (Figure 2b). The cumulative proportion surviving was lower in quartile 4 (FGF23 > 57.5 ng/L) (P < 0.05). The cumulative proportion surviving CVD death was highest in quartile 1 and lowest in quartile 4 (P < 0.05). The proportion surviving in quartiles 2 and 3 are intermediate, but their lines cross after about 3.5 years and the difference between quartile 1 and 3 becomes non-significant in the end.

In Cox proportional-hazard regression models analyzing all-cause mortality (Table 3) for quartiles of FGF23, and for log(10)FGF23, as a continuous variable there were no association after adjustment for eGFR and phosphate (only eGFR contributed to the decrease in HR). The association between log(10)PTH and all cause mortality in the unadjusted model waned after inclusion of eGFR. 25D was significantly associated with all-cause mortality with a reduction in risk of 10% (95% CI 5-10%)/10 nmol/L increase in 25D levels. Serum phosphate was not associated with mortality, HR (95% CI) were 1.75 (0.93 - 3.30)/mmol/L increase when adjusted for age.

**Table 3:** **Description of sub-cohorts with eGFR above and below 60 ml/min/1.73 m**^**2**^

**N = 2838**	**eGFR ≥60 ml/min/1.73 m**^**2**^	**eGFR <60 ml/min/1.73 m**^**2**^
	**N = 2075**	**N = 763**
**FGF23 Range (pg/ml)**	1.5 - 800	3.5 - 800
**FGF23 median (IQR)(pg/ml)**	40.3 (30–52)	54.1 (40–70)
**Age (years)**	75.1 ± 3.2	76.4 ± 2.9
**BMI (kg/m**^**2**^**)**	26.2 ± 3.5	27.0 ± 3.7
**Smoking (%)**	138 (7%)	96 (12%)
**Diabetes (%)**	174 (8%)	90 (12%)
**Hypertension (%)**	661 (32%)	360 (47%)
**Prevalent MI**	12%	20%
**Prevalent stroke**	5%	9%
**eGFR (ml/min/1.73 m**^**2**^**)**	78 (70–89)	50 (43–55)
**PTH median IQR (pmol/L)**	4.1 (2.8 - 5.5)	4.9 (3.4 - 6.9)
**Pi (mmol/L)**	1.07 ± 0.16	1.09 ± 0.18
**Ca (mmol/L)**	2.36 ± 0.15	2.36 ± 0.17
**Albumin (g/L)**	43.3 ± 3.3	42.8 ± 4.2
**25OHvitD (nmol/L)**	69.9 ± 23	69.7 ± 24
**Mortality rate/1000 py (95%CI)**	219/9458.4 py	134/3309.1
	23.2 (20.1 - 26.3)	40.5 (33.7 - 47.3)
**Cardiovascular deaths/1000 py (95%CI)**	73/9458.4 py	59/3309.1
	7.7 (5.9 - 9.5)	17.8 (13.3 - 22.3)

Analysing all factors in stepwise Cox regression revealed that age, BMI, albumin, calcium, eGFR, 25D, prevalent cancer and prevalent CVD were independently associated with all-cause mortality.

For CVD related death (Table 3) FGF23 was associated with increased HR between quartiles 1 and 2, and between 1 and 4. When adjusted for eGFR and phosphate the association became non-significant (P = 0.06 for Q1 vs. Q2), and when adjusted for the other parameters of mineral metabolism and prevalent co morbidities it became even weaker. When analyzed as a continuous variable the HR (95%) for CVD death was 26% (−1 to 59%) higher/(1-SD) increase in log(10)FGF23 in the multivariable model. The association between PTH and cardiovascular mortality did not remain when adjusted for eGFR and phosphate, and the HR (95%) for CVD related death was −6% (−14 to 2%)/10 nmol/L increase in 25D.

The sub-cohort with eGFR below 60 ml/min/1.73 m^2^ (N = 763, 26.9%) compared to the sub cohort with eGFR ≥ 60 ml/min/1.73 m^2^ had a mortality rate of 40.0 versus 23.1/1000 person-years (py), CVD mortality of 17.8 versus 7.7/1000 py, serum FGF23 of 54.1 (39.8 - 69.9) versus 40.3 (30.3 - 52.3) ng/L (Table 4).

**Table 4:** Cox regression for association between mortality of all-cause and parameters of mineral metabolism

**Factors**	**Model 1**	**Model 2**	**Model 3**
**All cause mortality HR (95%CI) FGF23**	**age**	**age, eGFR, phosphate**	**age, BMI, eGFR, albumin, calcium, phosphate, (PTH, FGF23 or 25D as appropriate), smoking, diabetes, hypertension, prevalent cancer, prevalent CVD**
Q1	ref	ref	ref
Q2	1.03	0.96	0.86
	(0.75 - 1.40)	(0.72 - 1.36)	(0.60 - 1.25)
Q3	0.93	0.85	0.82
	(0.67 - 1.29)	(0.61 - 1.38)	(0.58 - 1.16)
Q4	1.27	0.99	1.13
	(0.94 - 1.73)	(0.71 - 1.38)	(0.80 - 1.59)
Log10FGF23/(1-SD)	1.09	1.01	1.02
	0.97-1.22	0.89-1.14	0.89-1.17
Log PTH/(1-SD)	1.16 *	1.09	1.04
	(1.03 - 1.30)	(0.97 - 1.22)	(0.92 - 1.18)
25D/10 nmol/L	0.90 **	0.90 **	0.91 **
	(0.85 - 0.95)	(0.85 - 0.95)	(0.87 - 0.97)
CVD mortality HR (95%CI)			
Q1	ref	ref	ref
Q2	1.76 *	1.66	1.67
	(1.04 - 2.96)	(0.98 - 2.83)	(0.97 - 2.88)
Q3	1.15	1.00	0.80
	(0.64 - 2.07)	(0.55 - 1.82)	(0.42 - 1.51)
Q4	1.86 *	1.30	1.37
	(1.09 - 3.17)	(0.73 - 2.32)	(0.74 - 2.51)
Log10FGF23/(1-SD)	1.43 **	1.26*	1.26
	(1.18 - 1.73)	(1.01 - 1.56)	(0.99 - 1.59)
Log10PTH/(1-SD)	1.26 *	1.13	1.02
	(1.04 - 1.52)	(0.94 - 1.37)	(0.84 - 1.23)
25D/10 nmol/L	0.92 *	0.91 *	0.94
	(0.84 - 1.00)	(0.84 - 0.99)	(0.86 - 1.02)

The association between FGF23, PTH and 25D and prevalent CVD and HR for CVD mortality in those with eGFR above 60 ml/min/1.73 m^2^ (N = 2075, 218 deaths, 73 of CVD) are shown in Table 5. The HR (95% CI) for CVD mortality was 55% (13–111%)/(1-SD) increase in log(10)FGF23 in the multiadjusted model. PTH was not associated with increased risk for CVD death. In participants without prevalent CVD (N = 2324, 252 deaths, 75 of CVD) the HR (95% CI) for CVD death was 1.28 (0.95 - 1.56)/(1- SD) increase in log(10)FGF23.

**Table 5:** Cox regression models of parameters of mineral metabolism in sub-cohort with eGFR > 60 (N = 2075)

**Factors**	**Model 1 age**	**Model 2 age eGFR, phosphate**	**Model 3 age, BMI, eGFR, albumin, calcium, phosphate, (PTH, FGF23 or 25D as appropriate), smoking, diabetes, hypertension, prevalent cancer, prevalent CVD**
Mortality HR (95% CI)			
Log10FGF23/(1 - SD)	1.02	1.02	1.02
	(0.88 - 1.19)	(0.87 - 1.18)	(0.86 - 1.20)
Log10PTH/(1 - SD)	1.08	1.11	1.05
	(0.93 - 1.28)	(0.94 - 1.31)	(0.88 - 1.24)
25D/10 nmol/L	0.88	0.86	0.83
	(0.78 - 0.98)	(0.78 - 95)	(0.70 - 0.96)
Cardiovascular mortality HR (95% CI)			
Log10FGF23/(1-SD)	1.45*	1.44*	1.55*
	(1.11 - 1.89)	(1.09 - 1.89)	(1.13 - 2.11)
Log10PTH/(1-SD)	1.16	1.14	0.98
	(0.87 - 1.53)	(0.87 - 1.52)	(0.74 - 1.30)
25D/10 nmol/L	0.88*	0.88*	0.89
	(0.77 - 0.99)	(0.77 - 0.99)	(0.78 - 1.01)
Cox Hazards regression models of CVD-related death and parameters of mineral metabolism in the sub-cohort without prevalent cardiovascular disease			
No prevalent CVD (N = 2324)			
CVD mortality			
HR (95% CI)			
Log10FGF23/(1-SD)	1.37*	1.29	1.28
	(1.06 - 1.77)	(0.98 - 1.71)	(0.95 - 1.72)
Log10PTH/(1-SD)	1.16	1.11	1.10
	(0.89 - 1.52)	(0.85 - 1.44)	(0.85 - 1.43)
25D/10 nmol/L	0.92	0.92	0.94
	(0.82 - 1.03)	(0.82 - 1.03)	(0.84 - 1.05)

## Discussion

In this observational study of randomly selected elderly men the FGF23 level on inclusion was not associated with increased HR for mortality of all-cause. The HR (95% CI) for death due to CVD was 1.26 (0.99-1.59)/(1-SD) increase in log(10)FGF23 in the whole cohort, 1.55 (1.13 - 2.11) in those without CKD (eGFR > 60 ml/min/1.73 m^2^, and 1.28 (0.95-1.72) in those without known CVD on inclusion.

Several studies have demonstrated an independent association between FGF23 and mortality or cardiovascular events in CKD populations, but the association between FGF23 and mortality in non-CKD populations is not fully elucidated.

In a nested case–control study of incident haemodialysis patients *Gutierrez et al.* demonstrated an odds ratio (OR, 95% CI) of 5.7 (2.6 - 12.6) for death during first year on dialysis for the highest quartile of carboxy-terminal (C-)FGF23 (> 4010 RU/ml) compared to the lowest(<1090 RU/ml) [16].

In a study by *Isakova et al.* of 3879 CKD 2–4 patients from the Chronic Renal Insufficiency Cohort (CRIC) followed for in median 3.5 years there were 266 deaths. FGF23 at base line was associated with 50% (95% CI: 30–70%) increase in death/unit increase in logFGF23 [6]. *Kendrick et al.* examined 1099 CKD 4–5 patients and found an association between higher FGF23 and mortality risk [17]. *Seiler et al.* found in a cohort of CKD patients (N = 149, eGFR = 36 ± 23 ml/min/1.73 m^2^) that baseline FGF23 predicted future cardiovascular complications [18].

There are also a few studies of FGF23 and mortality in non-CKD populations. *Parker et al.* described in a cohort of 833 persons, with prevalent stable coronary disease, a HR (95% CI) for death of 40% (22–60%)/doubling of FGF23. The majority of deaths were due to cardiovascular disease and there was an association between FGF23 and heart failure and stroke, but not with myocardial infarction [11].

*Taylor et al.* made a nested case–control study from a large cohort of healthy males (age 63.6 ± 8.6 years, creatinine 1.0 ± 0.2 mg/dL). During 10 years follow-up 422 developed myocardial infarction or died of coronary heart disease. Cases did not differ from the 837 controls concerning C-FGF23 58.6 (47.8 - 73.7) vs. 57.1 (47.4 - 72.2) RU/ml. or PTH, while 25D was significantly lower in cases [19].

*Ix et al.* reported of the association between C-FGF23 and death, heart failure and CVD in the general population. They followed 3017 persons 65 years or older (mean age 78 ± 5 years), without active cancer treatment, for in median 10.5 years and 1730 deaths occurred. They found a HR (95% CI) for all cause death of 25% (14–36%)/doubling of C-FGF23, incident heart failure of 41% (23–61%) and of incident CVD of 12% (−2%-29%) [20].

A recent report of a community derived cohort of elderly men describes an association between intact FGF23 and all-cause and cardiovascular mortality. The effect is mainly driven by the strong increase in risk for CVD death among the quintile with highest FGF23 (> 60 ng/L), and among individuals with decreased renal function. That study also indicate that a very low FGF23 is associated with the lowest risk for CVD death [21].

Our study, on the other hand, does not demonstrate an association between FGF23 and all-cause death. We study a homogenous group of randomly selected voluntary Swedish males, avoiding bias due to gender or ethnicity, but probably introducing bias to more health interested and active persons. The mortality rate due to CVD was only 37% of total mortality rate, which may explain the negative result between FGF23 and all-cause mortality. In crude analysis there is an association between FGF23 and CVD mortality, but the relationship is not linear when analyzed in quartiles and the increased risk in quartile 4 (FGF23 > 57 ng/L) vanishes after adjustment for eGFR. Though, renal dysfunction explains most, or all, of the association between FGF23 and CVD mortality. Analyzed as a continuous variable there is a 25% increase in HR for CVD death/(1 - SD) increase in Log10FGF23, but the formal significance is borderline (P = 0.06). The association in the sub cohort with eGFR above 60 ml/min/1.73 m^2^ remained significant after adjustment, Death due to CVD in this study includes heart failure and stroke, but also myocardial infarction, pulmonary embolism and arrhythmias for which the association may be weaker or absent, in accordance with the other studies. One explanation for the divergent findings may be that eGFR is associated with increased mortality and with increased FGF23. In CKD populations FGF23 levels may indicate increased risk due to duration of decreased renal function, phosphate load or specific renal injury, while in a rather healthy population where decreased GFR mostly is due to prevalent CVD FGF23 may not add much information about mortality risk.

When excluding those with decreased eGFR FGF23 still is associated with a modest increase in HR for CVD death. The explanation may be that lower FGF23 signals better renal function, lower BMI and maybe lower intake of phosphate creating a low risk profile for CVD not fully adjusted for in the models.

Phosphate was not associated with mortality or even with FGF23 in this study. That may be explained by the generally good renal function. Higher phosphate would lead to higher FGF23 but as long as renal function is able to increase the fractional excretion of phosphate in response to FGF23, and PTH, normophospahtemia will prevail.

In several earlier studies FGF23 was analyzed with an assay detecting intact and carboxy-terminal FGF23 (C-FGF23), which has been shown to correlate strongly with intact FGF23 independently of GFR. We measured intact FGF23 in serum since at the time of analysis there was concern for accumulation of C-terminal fragments as renal function declined. Recent reports suggest instability of the intact FGF23 molecule in vitro and rather underestimation of circulating FGF23, but immediate centrifugation and freezing should minimize this source of attenuation of effect. We do not believe the choice of assay caused the lack of association with mortality in our study, but it cannot be excluded, and direct comparisons of different FGF23 assays will be necessary in the future.

We also report the lack of an association between PTH and HR for all-cause and CVD death after adjustment for eGFR.

Higher 25D levels was associated with lower HR for death of all-cause even after adjustments, which has been described in detail in this cohort earlier [22].

Strengths of this study are that it describes a large community-derived cohort with a relatively large number of events and complete data of survival. The population is homogenous concerning gender and ethnicity. Renal function is evaluated with a cystatin C based formula. The Swedish official cause of death register is comprehensive and reliable

There are also several limitations. It is not possible to generalize the results to females, other age-groups or ethnicities. Even if the vital status of the subjects were certain, we used the cause of death code from the National cause of death register and they were not adjudicated by clinicians. Differentiating between CVD related causes of death would have been informative, since the putative mechanism would explain an association with heart failure rather than acute coronary syndromes. Data about prevalent disease were provided by the subjects and may have caused misclassifications. FGF23 was measured as single measurements. These limitations may lead to an underestimation of the association between FGF23 and outcome. 1,25 dihydroxy vitamin D levels, lipid levels and other known CVD risk markers were not available in this study. We also lacked data of phosphate intake and renal phosphate excretion.

## Conclusion

In the general population there is no association between FGF23 levels and death of all-cause independent of eGFR. There is a possible association between higher FGF23 and CVD deaths in individuals with normal renal function. Higher FGF23 is associated with CVD death due to decreased renal function, while the lowest FGF23 levels may indicate a low risk profile for CVD disease.

## Competing interests

The authors declare that they have no competing interests.

## Authors’ contributions

PAW made calculations and wrote the manuscript, ÅT was responsible collection of mortality data, MKK for handling the MrOS data in Malmoe, CO and DM for handling the MrOS data in Gothenburg, EO for coordinating the international MrOS, TEL for FGF23 analyses in MrOS, TL for planning the study and ÖL for MrOS in Uppsala and planning the study. All authors have read and approved of the contents of the manuscript.

## Pre-publication history

The pre-publication history for this paper can be accessed here:

http://www.biomedcentral.com/1471-2369/14/85/prepub
